# Nonkeratinocyte skin cancer in survivors of childhood, adolescent, and young adult cancer with and without radiotherapy

**DOI:** 10.1016/j.jdin.2025.09.019

**Published:** 2025-10-14

**Authors:** Yuming Sun, Zehao Luo, Furong Zeng, Guangtong Deng

**Affiliations:** a Department of Dermatology, Xiangya Hospital, Central South University, Changsha, China; b Furong Laboratory, Changsha, China; c Department of Plastic and Cosmetic Surgery, Xiangya Hospital, Central South University, Changsha, China; d Department of Oncology, Xiangya Hospital, Central South University, Changsha, China

**Keywords:** adolescent and young adult, childhood, nonkeratinocyte skin cancer, radiotherapy, survivors

*To the Editor:* Childhood cancer survivors exhibit an elevated risk of developing primary keratinocyte carcinomas with or without radiotherapy.^1^ Here, we present an analysis on the risk of developing nonkeratinocyte skin cancer in survivors of childhood, adolescent, and young adult cancer (CAYAC) with or without radiotherapy based on the latest Surveillance, Epidemiology, and End Results (SEER) Program.

An initial CAYAC diagnosis and a subsequent nonkeratinocyte skin cancer diagnosis was determined using the International Classification of Diseases for Oncology, Third Edition.^2^ Patients with CAYAC were defined as cancer survivors aged 0 to 39 years, encompassing both childhood cancer survivors aged 0 to 19 years and adolescent and young adult cancer survivors aged 15 to 39 years.^1^^,^^3^ Nonkeratinocyte skin cancer referred to skin cancer except squamous or basal cell cancer, mainly including melanoma, Merkel cell carcinoma, adnexal cancers, cutaneous sarcomas, and cutaneous lymphoma.^2^ We collected data on the patients’ age at diagnosis, gender, race, calendar year of primary cancer diagnosis, and follow-up since primary cancer, stratified by radiotherapy status. We assess the risk of developing subsequent nonkeratinocyte skin cancer in CAYAC survivors by standardized incidence ratios (SIRs) with 95% confidence intervals (CIs). We also computed the excess risks per 100,000 person-years. Nonkeratinocyte skin cancer arising within 2 months of an initial diagnosis of CAYAC were considered synchronous tumors and were excluded by SEER∗Stat.^4^

A total of 87,702 CAYAC survivors were included in the study (Supplementary Material, available via Mendeley at https://data.mendeley.com/datasets/s6h8kxr6cg/1). Among them, 21,754 (24.8%) received radiotherapy, and the basic characteristics are shown in Table I. There were 277 subsequent nonkeratinocyte skin cancer among CAYAC survivors, including melanoma (208 [75.1%]), Merkel cell carcinoma (2 [0.7%]), adnexal cancers (10 [3.6%]), cutaneous sarcomas (22 [7.9%]), and cutaneous lymphoma (35 [12.6%]). We observed an increased incidence rate of subsequent nonkeratinocyte skin cancer. It was 122% higher in CAYAC survivors without or unknown radiotherapy (SIR, 2.22 [95% CI, 1.91-2.56]) and 204% higher in those with radiotherapy (SIR, 3.04 [95% CI, 2.46-3.71]), compared with their corresponding general population. Among CAYAC survivors without or unknown radiotherapy, elevated risks were observed for melanoma (SIR, 1.84 [95% CI, 1.54-2.18]), Merkel cell carcinoma (SIR, 8.43 [95% CI, 1.02-30.45]), adnexal cancers (SIR, 4.04 [95% CI, 1.10-10.35]), cutaneous sarcomas (SIR, 3.10 [95% CI, 1.84-4.90]), and cutaneous lymphoma (SIR, 7.45 [95% CI, 4.86-10.91]), whereas significantly increased risk of melanoma (SIR, 2.77 [95% CI, 2.19-3.47]) and adnexal cancers (SIR, 16.45[95% CI, 6.04-35.81]) were observed in those who received radiotherapy (Table II). We also performed sensitivity analyses in adolescent and young adult cancer survivors, and observed consistent results (Supplementary Material, available via Mendeley at https://data.mendeley.com/datasets/s6h8kxr6cg/1).

**Table I:** Characteristics of childhood, adolescence, and young adult cancer survivors by radiotherapy

Characteristic	Total, no. (%)	No/unknown RT, no. (%)	RT, no. (%)
Total	87,702	65,948	21,754
Age at diagnosis, y			
0-14	27,434 (31.3)	22,002 (33.4)	5432 (25.0)
15-39	60,268 (68.7)	43,946 (66.6)	16,322 (75.0)
Gender			
Female	39,659 (45.2)	29,846 (45.3)	9813 (45.1)
Male	48,043 (54.8)	36,102 (54.7)	11,941 (54.9)
Race∗			
White	70,511 (80.4)	52,821 (80.1)	17,690 (81.3)
Black	9710 (11.1)	7599 (11.5)	2111 (9.7)
Other	7481 (8.5)	5528 (8.4)	1953 (9.0)
Calendar year of first primary cancer diagnosis			
2000-2005	29,458 (33.6)	21,186 (32.1)	8272 (38.0)
2006-2010	26,445 (30.2)	19,808 (30.0)	6637 (30.5)
2011-2016	31,799 (36.3)	24,954 (37.8)	6845 (31.5)
Months of follow-up since first primary cancer			
60-119	32,054 (36.5)	24,771 (37.6)	7283 (33.5)
120-179	26,082 (29.7)	19,690 (29.9)	6392 (29.4)
≥180	29,566 (33.7)	21,487 (32.6)	8079 (37.1)
Primary malignancy			
Bones and joints	4069 (4.6)	3331 (5.1)	738 (3.4)
Brain/CNS tumors	15,430 (17.6)	9986 (15.1)	5444 (25.0)
HL	16,894 (19.3)	10,164 (15.4)	6730 (30.9)
Kidney and renal pelvis	8115 (9.3)	7198 (10.9)	917 (4.2)
Leukemia	20,546 (23.4)	18,550 (28.1)	1996 (9.2)
NHL	15,918 (18.2)	12,421 (18.8)	3497 (16.1)
Soft tissue including heart	6730 (7.7)	4298 (6.5)	2432 (11.2)

*CNS*, Central nervous system; *HL*, Hodgkin lymphoma; *NHL*, non-Hodgkin lymphoma; *RT*, radiotherapy.

∗ Other races include Asian or Pacific Islander, American Indian/Alaska Native.

**Table II:** SIRs of subsequent Nonkeratinocyte skin cancers among childhood, adolescence, and young adult cancer survivors stratified by radiotherapy

Nonkeratinocyte skin cancer type	Observed	Expected	SIR (95% CI)∗	ER	OIR	EIR
All						
No/unknown RT	181	81.67	2.22 (1.91-2.56)†	1.23	22.34	10.08
Radiation received	96	31.57	3.04 (2.46-3.71)†	2.31	34.49	11.34
Melanoma of the skin						
No/unknown RT	131	71.15	1.84 (1.54-2.18)†	0.74	16.17	8.78
Radiation received	77	27.76	2.77 (2.19-3.47)†	1.77	27.67	9.97
None melanoma‡						
No/unknown RT	50	10.53	4.75 (3.53-6.26)†	0.49	6.17	1.3
Radiation received	19	3.81	4.98 (3.00-7.78)†	0.55	6.83	1.37
Merkel cell carcinoma§						
No/unknown RT	2	0.24	8.43 (1.02-30.45)†	0.02	0.25	0.03
Radiation received	-	-	-	-	-	-
Adnexal cancers						
No/unknown RT	4	0.99	4.04 (1.1-10.35)†	0.04	0.49	0.12
Radiation received	6	0.36	16.45 (6.04-35.81)†	0.2	2.16	0.13
Sarcomas						
No/unknown RT	18	5.81	3.10 (1.84-4.90)†	0.15	2.22	0.72
Radiation received	4	2.11	1.90 (0.52-4.86)	0.07	1.44	0.76
Lymphoma						
No/unknown RT	26	3.49	7.45 (4.86-10.91)†	0.28	3.21	0.43
Radiation received	9	1.25	7.18 (3.29-13.64)†	0.28	3.23	0.45

*EIR*, Expected incidence rate per 100,000 persons/y; *ER*, excess risk per 100,000 persons/y; *OIR*, observed incidence rate per 100,000 persons/y; *RT*, radiotherapy; *SIR*, standardized incidence ratios.

∗ SIR indicates the ratio of observed cases of subsequent primary cancers to the expected number in the general population, adjusted for age, sex, race, and year of cancer diagnosis.

† *P* < .05, SEER∗Stat indicates statistical significance (*P* < .05) but does not report exact *P* values.

‡ Includes Merkel cell carcinoma, adnexal cancers, cutaneous sarcomas, and cutaneous lymphomas.

§ No patients received radiotherapy.

With improvements in detection, treatment, and supportive care, the survival rate of patients with cancer has increased, drawing increasing attention about long-term adverse events, especially in CAYAC survivors.^1^ Our findings indicate an elevated risk of nonkeratinocyte skin cancers among CAYAC survivors, with markedly increased risks of melanoma and adnexal cancers in those receiving radiotherapy; however, the causality from the radiation still needs to be elucidated.^5^ Limitations include uncontrolled confounders such as family history, genetic factors, sun exposure, and short latency time, along with a small sample size of nonkeratinocyte skin cancer cases beyond melanoma, requiring further analysis.

## Conflicts of interest

None disclosed.
